# Application of Non-Aflatoxigenic *Aspergillus flavus* for the Biological Control of Aflatoxin Contamination in China

**DOI:** 10.3390/toxins14100681

**Published:** 2022-09-30

**Authors:** Wan Zhang, Jianpeng Dou, Zidan Wu, Qiu Li, Shanshan Wang, Huiru Xu, Wenfu Wu, Changpo Sun

**Affiliations:** 1 Department of Biological and Agricultural Engineering, Jilin University, Changchun 130022, China; 2 Institute of Grain and Oil Science and Technology, Academy of National Food and Strategic Reserves Administration, Beijing 100037, China; 3 Shandong Luhua Group Co., Ltd., Yantai 265200, China

**Keywords:** aflatoxin, biological control, non-aflatoxigenic, *Aspergillus flavus*, peanut, rice

## Abstract

Biological control through the application of competitive non-aflatoxigenic *Aspergillus flavus* (*A. flavus*) to the soil during peanut growth is a practical method for controlling aflatoxin contamination. However, appropriate materials need to be found to reduce the cost of biocontrol products. In this study, a two-year experiment was conducted under field conditions in China, using a native non-aflatoxigenic strain to explore its effect. After three months of storage under high humidity, aflatoxin levels remained low in peanuts from fields treated with the biocontrol agent. Three types of substrates were tested with the biocontrol agent: rice grains, peanut meal (peanut meal fertilizer) and peanut coating. Compared to untreated fields, these formulations resulted in reductions of 78.23%, 67.54% and 38.48%, respectively. Furthermore, the ratios of non-aflatoxigenic *A. flavus* recovered in the soils at harvest in the treated fields were between 41.11% and 96.67% higher than that in untreated fields (25.00%), indicating that the rice inoculum was the most effective, followed by the peanut meal fertilizer and peanut coating. In 2019, the mean aflatoxin content of freshly harvested peanuts in untreated fields was 19.35 µg/kg higher than that in the fields treated with 7.5 kg/ha rice inoculum, which was 1.37 µg/kg. Moreover, no aflatoxin was detected in the two other plots treated with 10 and 15 kg/ha rice inoculum. This study showed that the native Chinese non-aflatoxigenic strain of *A. flavus* (18PAsp-zy1) had the potential to reduce aflatoxin contamination in peanuts. In addition, peanut meal can be used as an alternative substrate to replace traditional grains, reducing the cost of biocontrol products.

## 1. Introduction

Peanuts growing in soil are at risk of infection by aflatoxin-producing *Aspergillus* species such as *A. flavus* and *A. parasiticus* [1]. Aflatoxin contamination in peanuts often occurs more frequently when they are stressed by high temperatures and dry conditions during pod maturation (pre-harvest) [2] or unfavorable storage conditions (post-harvest) [3]. Aflatoxins are carcinogenic, and aflatoxin B_1_ (AFB_1_) is a powerful liver carcinogen [4,5]. Additionally, aflatoxin contamination may influence the quality of crops, such as peanuts and maize, causing substantial economic losses. Many countries have set strict standards for these agricultural products. The European Union (EU) set the upper limit of AFB_1_ in peanuts to be 2 µg/kg, and the total aflatoxin content (AFG_2_ + AFG_1_ + AFB_2_ + AFB_1_) to be 4 µg/kg [6,7].

Humans and animals cannot degrade or remove aflatoxins from the body [8]. Moreover, the aflatoxin temperature threshold can reach 268 °C, so these toxins are resistant to high temperatures [9,10]. The adsorption method cannot fundamentally eliminate their toxicity [11]. Enzymatic hydrolysis may affect the quality of peanuts or peanut products to some extent; for instance, it will adversely affect the flavor and quality of peanut oil [12]. Therefore, it is important to prevent the production and/or ingestion of aflatoxins. Biological control (biocontrol) implemented with proper agricultural practice and the breeding of peanut varieties resistant to *A. flavus* are useful in preventing and reducing aflatoxin contamination [13]. A 2017 study reported that non-aflatoxigenic (non-AF) *A. flavus* was the preferred agent for biocontrol formulations for peanuts [1]. Weaver and Abbas [14] proved that the applied biocontrol strains of non-AF *A. flavus* had potential to displace the indigenous aflatoxigenic (AF) strains. Additionally, strains screened from different regions may have adaptive characteristics. Therefore, the non-AF strains used as biocontrol agents should be isolated from the soil where they will be applied, and they should have high competitiveness [1,15]. It is the geographic and strain specificity that drives the insistent search for better biocontrol of aflatoxins worldwide.

The biocontrol method has been used in many countries. As early as 1990, Cotty et al. applied non-AF *A. flavus* to cotton fields, effectively reducing aflatoxin pollution in cotton seeds [16]. In 1992, Dorner et al. proposed that the application of non-AF *A. parasiticus* to soil used for peanut planting could reduce aflatoxin pollution by 83–85% [17]. Several countries have registered commercial strains of *A. flavus*. Two products, named Afla-Guard^®^ and AF36^®^, registered in the United States in 2004, were the earliest commercially available non-AF *A. flavus* [18]. Afla-Guard^®^ is mainly used to control aflatoxins in maize and peanuts, while AF36^®^ is mainly used to control aflatoxins in crops such as cotton, almonds, maize, figs and pistachios [1]. In several African countries, multiple non-AF *A. flavus* strains have been identified to competitively inhibit toxin-producing strains in maize and peanut fields. These non-AF strains have achieved good results in laboratory and field tests, reducing aflatoxin contamination by 70% to 99% [19]. They have been combined into region-specific versions of Aflasafe^TM^, a product comprised of four strains of non-AF *A. flavus*, which are commercially available throughout Africa [20]. In Italy, a biocontrol formulation involving non-AF strain MUCL54911 has been registered for use with maize [21]. In China, research on the screening of non-AF strains and in vitro experiments in the laboratory have progressed. For example, a highly efficient and competitive non-AF *A. flavus* strain, AF051, with a large deletion (89.59 kb) in its aflatoxin gene cluster was isolated from Jiangsu Province [22]. Another group isolated a non-AF *A. flavus* strain from Henan Province that effectively inhibited growth of AF *A. flavus* [23]. However, there are few reports related to field experiments on aflatoxins in peanuts in China.

At present, the commonly used method for the preparation of formulation is to coat grains such as sorghum, barley or wheat with the spore suspension of the non-AF agent as a carbon source to give it an advantage over indigenous (toxigenic) strains on the soil environment [24]. However, this method is more suitable for big companies to conduct large-scale industrial applications, because it is too expensive for farmers. Therefore, it is important to create more cost-efficient biocontrol formulations or to reduce the amounts of agents by promoting their long-term residence [25]. To this end, scientists have conducted extensive exploration. For example, Accinelli et al. used bioplastic-based formulations to introduce non-AF *A. flavus* into the field, achieving good colonization of non-AF strains and desirable biocontrol effects [26,27].

In this paper, a non-AF *A. flavus* strain from Henan Province with effective competitiveness traits was tested as a potential biocontrol agent in a two-year peanut study. In 2018, field experiments were conducted in Henan and Hubei Provinces, which have similar climates and soil conditions, testing three alterative carbon sources: peanut meal, rice grains and a peanut coating agent (i.e., water-soluble starch, sodium alginate and glycerol). In 2019, a larger field experiment in Henan Province was conducted using different quantities of rice inoculum alone. The goals of this study were to (1) explore the potential of the *A. flavus* strain to prevent aflatoxin contamination and (2) find an alternative substrate that offered a comparable carbon source at a reduced cost to growers.

## 2. Results

### 2.1. Peanut Yield in Each Biocontrol-Treated Plot

Within each province in 2018, no significant yield differences among treatments by carbon source were observed (*p* > 0.05). However, the peanut yield in Henan Province was higher than that in Hubei Province, with averages of 945.32 and 809.44 kg, respectively. The average yield of peanut in the plots with low, medium and high rice inoculum applied in 2019 was 424.23, 446.37 and 455.07 kg, respectively. Peanut yields in 2019 were lower than those of the 2018 rice inoculum plots, probably due to climatic effects.

### 2.2. Distribution of Aspergillus flavus in Soils

There was no significant difference in the field density of *A. flavus* and the proportion of non-AF *A. flavus* in the soil of each plot before planting, nor between the treated or untreated plots at harvest (Table 1 and Table 2). For instance, out of 3.49 logCFU/g isolates recovered from soil prior to planting in Henan Province in 2018, 31.11% were non-AF isolates (Table 1). Additionally, there was no significant increase in the proportion of non-AF *A. flavus* strains in the soil at harvest in the control plots. As expected, the application of each biocontrol formulation in the 2018 and 2019 studies increased the soil abundance of non-AF *A. flavus* isolated at harvest compared to those before planting. Additionally, in most instances, the frequencies of non-AF strains in treated soils at harvest were significantly (*p* < 0.05) higher than those in corresponding untreated soils in both provinces (Table 1). In Hubei Province, for example, frequencies of non-AF *A. flavus* in soils from treated fields ranged from 41.11% in plots treated with the peanut coating agent to 94.44% in plots treated with the rice inoculum (Table 1). Similarly, compared to untreated samples, significantly (*p* < 0.05) higher frequencies of non-AF *A. flavus* were measured in peanut soils at harvest in plots treated with the three types of formulations in Henan Province. These frequencies were significantly (*p* < 0.05) higher than those in soils from corresponding untreated fields, i.e., 23.33% and 26.67% in Henan Province and Hubei Province, respectively (Table 1). However, in most of the soils at harvest, apart from plots treated with the peanut meal in Hubei Province, there were no significant differences (LSD, *p* > 0.05) in the frequencies of non-AF *A. flavus* between the groups subjected to the high- and low-dose treatments (Table 1). With respect to treated samples, incidences of non-AF *A. flavus* were recorded in the three groups in the following descending order: rice inoculum, peanut meal and peanut coating.

The proportion of *A. flavus* and non-AF *A. flavus* strains at harvest was significantly higher (*p* < 0.05) in the soil of plots applied with rice inoculum than in the untreated plots in 2019. The density of *A. flavus* and the proportion of non-AF *A. flavus* strains at harvest were higher in the plots with medium and high doses of rice inoculum than in the plots with low doses, but there was no significant difference between medium and high doses (*p* > 0.05). The rice inoculum in 2019 achieved the same effect as in 2018, i.e., elevated the density of *A. flavus* in the soil, while increasing the proportion of non-AF *A. flavus*. This indicated that the rice inoculum application method remained effective in the enlarged test field. There was no significant difference between the high dose group that applied 15 kg/ha and the medium dose group that applied 10 kg/ha, indicating that the dosage of 10 kg/ha can meet the needs of biological control in peanut fields.

### 2.3. Aflatoxin Concentrations in Treated and Untreated Peanut Kernels

In 2018, the average moisture content of freshly harvested peanuts was 40.14%. Seed moisture content can affect aflatoxin concentration assessments, and too much moisture will affect the crushing effect of peanut kernels. Therefore, before the extraction of aflatoxins, the average moisture content was reduced to 1.74%. Aflatoxins were not detected in most of the peanuts immediately after harvest and after three months of storage under normal conditions in both provinces; aflatoxins were only detected (avg. range = 3.03 μg/kg (ppb) total aflatoxins) in untreated peanuts immediately after harvest in Hubei Province in 2018. However, aflatoxins were detected in peanut kernels collected from every field after three months of storage at more than 90% humidity in both provinces (Table 3).

The average total aflatoxin B_1_ (AFB_1_) content of untreated peanuts was 41.35 ppb in Henan Province and 62.29 ppb in Hubei Province (Table 3). Treatment of peanuts with rice inoculum, peanut meal and peanut coating, apart from the high-dose peanut-coated group in Hubei Province, resulted in significantly (*p* < 0.05) less (44.33% to 81.90%) AFB_1_ compared to untreated peanuts in both provinces (Table 3). There was no significant difference between the low- and high-dose plots with respect to the reduction of AFB_1_ (LSD, *p* > 0.05).

In 2019, aflatoxins were detected in the untreated field and the fields treated with 7.5 kg/ha biocontrol agents; the average values were 19.35 ppb and 1.37 ppb, respectively. Aflatoxin was not detected in other treated fields. The reduction of the aflatoxin content in the treated fields was 100% for the 10 and 15 kg/ha rates and 92.92% for the 7.5 kg/ha rate.

## 3. Discussion

The studies described here are the first to examine the use of non-AF biocontrol agents based on rice and peanut meal under field conditions in China. The non-AF *A. flavus* strain, 18PAsp-zy1, was obtained from Henan Province in China [23]. Both the 2018 and 2019 field trials were carried out in Henan Province, so the biocontrol strain was indigenous to the local environment [28,29]. Previously, it was determined that the reason for the inability of 18PAsp-zy1 to produce aflatoxin was a mutation in its *aflR* promoter sequence [23]. This mutation distinguishes our biocontrol strain from other non-AF strains used as biocontrol. For example, NRRL 21882, which is used as an active ingredient in the biocontrol product Afla-Guard^®^, has a nearly 80 kb deletion of the entire aflatoxin gene cluster [30]. NRRL 18543 (AF36) has a frameshift mutation elsewhere in its aflatoxin gene cluster, *pksA* or *aflC*, which has been associated with its inability to produce aflatoxin [31,32]. The 18PAsp-zy1 strain used in this paper inhibited 72.6% of AFB_1_ production in vitro [23]. Under field conditions, it was known that 74–100% of AFB_1_ production could be inhibited based on two years of data (Table 3). NRRL 21882 inhibited 70–90% of AFB_1_ production in peanuts in field experiments [33,34]. An important reason for the competition between non-AF and AF strains is that their growth requires similar nutrients [35]. However, competitiveness is considered key to *A. flavus* biocontrol success. Among biocontrol strains, the genotype of non-AF strain that confers the best competitive advantage remains unclear. Does having a complete aflatoxin gene cluster with a single point mutation in a single gene offer an advantage? Or does a lack of these pathway genes altogether offer an advantage? This needs to be explored further.

Peanuts are susceptible to aflatoxin contamination, which is produced by AF *A. flavus* found in the soil [1]. In order to control aflatoxin contamination in peanuts at source, it is necessary to prevent the infestation of peanuts by AF *A. flavus*, which can be achieved by enhancing the proportion of non-AF strains in the soil. In this paper, three types of formulations were prepared. When the non-AF *A. flavus* spores were applied to the field, they would go through the stages of germination, growth and reproduction. The more nutrients they received during this period, the longer they persisted in the field. Therefore, the persistence of non-AF strains in the soil was important and directly affected their inhibitory effect on the AF strains. In this paper, the proportion of non-AF *A.* *flavus* in the soil of plots with different formulations applied at harvest were examined and results showed that the average proportions of non-AF strains in the experimental plots with rice inoculum, peanut meal fertilizer and peanut coating agent were 95.00%, 69.72% and 41.94%, respectively (Table 1). This indicated that the biocontrol strain preferably colonized the rice grain as the carbon source, which correlated with its persistence in the soil.

Moreover, no aflatoxin was detected after three months of normal storage, which suggested good post-harvest aflatoxin control. Alternatively, the lack of detectable aflatoxin could relate to the water content in the stored peanuts, which was nearly 3% lower than the safe storage content of peanuts (9%), after exposure to sunshine for five days that prevented *A. flavus* from infecting the peanuts [36]. Low water activity is inhibitory to growth of *A. flavus* and *A. parasiticus* [37]. After three months of storage under high humidity, aflatoxins were detected in samples from every plot, but biocontrol-induced reductions (ranging from 13.15% to 81.90%; Table 3) were observed in samples from treated plots suggesting a continued biocontrol effect during storage. Dorner et al. [38] found the average aflatoxin content in stored peanuts that had been treated with biocontrol during their growing season (pre-storage) was 95.9% lower than in peanuts without field treatment with biocontrol. Furthermore, experimental comparisons were conducted and found that the application of biocontrol agents in the field was better than the application of microbial agents just before storage.

The preparation of inoculum using grains (i.e., rice, sorghum, or barley grains) as a carrier, which can provide sufficient nutrients for spores and facilitate their colonization in the soil, is now relatively common worldwide [39]. However, considering the cost of the formulation, it is better to find other substrates [25]. This is particularly important for farmers who manage their fields themselves; for example, in China a majority of peanut fields are managed by farmers themselves instead of companies [40]. Cassava peels had been tested to determine whether they were suitable to replace grains in preparing biocontrol products in the laboratory. However, fewer spores were obtained due to insufficient protein content, fatty acids and minerals [41]. To reduce the biocontrol cost and effectively use peanut byproducts, this study used peanut meal and liquid seed coating as substrates; the peanut meal improved the ratio of non-AF *A. flavus* strains in soils at harvest to an average of 69.72% and reduced aflatoxin contamination by 67.54% in peanuts during storage compared with untreated fields (Table 1 and Table 3). Additionally, peanut meal, as the byproduct of peanut oil, is also a good source of plant protein and contains high concentrations of energy components [42], having been used as animal feed (i.e., fish and pig) [43,44]. This is the first time that peanut meal has been used as the substrate to make biocontrol products for aflatoxin contamination in peanut fields. In 2019, China’s peanut planting area was 4.6 million ha, accounting for 17.5% of the global area, and the total output was 17.5 million tons, approximately 52% of which was used for oil extraction, accounting for 39.4% of the global amount [40]. Reasonable use of peanut meal can effectively improve the utilization of agricultural resources. Additionally, peanut meal has good heat dissipation properties, and it can be used to effectively control temperature during the fermentation process [45]. However, due to its high nutritional content, it is necessary to prevent contamination by other bacteria during fermentation.

The experimental results were slightly different between the two years. In 2018, no aflatoxin was detected in the freshly harvested peanuts, regardless of treatment. However, in 2019, the reduction of aflatoxin content of freshly harvested peanuts in the fields treated with the three doses of rice inoculum ranged from 92.92% to 100% compared with the untreated fields, and the aflatoxin content of the peanuts in the treated plots satisfied the EU standard (2 μg/kg) [7]. The main reason for this result should be the climate. The weather in 2018 was milder than that in 2019. These results are not uncommon. Zanon et al. [46] performed a two-year biocontrol study of aflatoxins in peanut fields in Argentina. Aflatoxins were detected in peanut kernels harvested under drought stress in the second year. However, aflatoxins in peanuts in other plots without drought stress were not detected. The results of aflatoxins in peanuts were similar to those obtained in a field experiment conducted by Weaver et al. from 2012 to 2015 in Washington County, Mississippi, in which the toxin content in corn was not significantly different between untreated and treated fields (only a few samples of corn were detected, and sometimes the concentrations were almost undetectable) [14].

In the current study, the distribution of *A. flavus* was significantly different in the soils at harvest between the untreated and treated fields (LSD, *p* < 0.05, Table 1). It appears that the structure of the colonies in the soils was influenced [47]. The density of *A. flavus* in the untreated fields increased in harvest compared with that before planting, and was even higher than that in some treated fields. For instance, the density of *A. flavus* in untreated fields in Hubei Province in 2018 was 4.06 logCFU/g higher than that in fields treated with the peanut meal and peanut coating (Table 1). This may have been due to the increase in temperature and the increase in moisture in the soil with rainfall, causing *A. flavus* spores to germinate, grow and expand, so the total number of *A. flavus* colonies in the soil increased. However, the proportion of non-AF strains in the untreated fields did not increase. In contrast, the proportion of AF strains in the treated fields significantly increased. The soil is the main niche where *Aspergillus* strains infect peanuts. It seems that if the ratio of AF to non-AF strains in soil changes, the strains in peanuts would also change [14].

## 4. Conclusions

18PAsp-zy1 is the first native non-AF strain from China to be field tested as an active ingredient to significantly reduce aflatoxin in peanuts. In 2018, the three different formulation types with 18PAsp-zy1 as the biocontrol agent showed effectiveness at reducing aflatoxin contamination in peanuts, with rice inoculum offering the greatest biocontrol persistence and aflatoxin control. This was confirmed in 2019 while testing rice inoculum alone. However, peanut meal as a carbon source was also suitable. This type of formulation needs continued verification through more field experiments, since use of peanut meal offers two benefits to growers: less peanut waste and, therefore, less expense to growers.

## 5. Materials and Methods

### 5.1. Strain Selection

The non-AF strain used was an *A. flavus* strain named 18PAsp-zy1, which is a naturally occurring isolate obtained from a peanut field in Zhengyang City in Henan Province, China. In a previous study, this strain was shown to lack production of aflatoxin and cyclopiazonic acid. Additionally, the strain was confirmed to have a good inhibitory effect on AF *A. flavus* [23].

### 5.2. Biocontrol Formulation Preparation and Application

Three types of formulations were developed using different carbon sources for the biocontrol strain to colonize: rice grains, peanut meal and a peanut coating agent. The non-AF *A. flavus* was removed from storage at −80 °C and activated on potato dextrose agar (PDA) plates for three days at 30 °C. 

Rice inoculated with spores was produced with a method modified from Zanon et al. [46]. The spores of non-AF *A. flavus* were prepared using soybean culture medium, and then they were harvested using plant oil. The suspension of soybean oil-dissolved non-AF *A. flavus* spores was mixed with rice, and then 2% *w*/*w* of diatomite was added to disperse the spores on the rice surface. The concentration of the spores on the rice inoculum reached 10^8^ CFU/g for low dose in 2018 and all doses in 2019. The concentration of the spores on the rice inoculum reached 10^9^ CFU/g for high dose in 2018. The rice inoculum was stored in the laboratory at room temperature and kept in a sealed place.

The spores of non-AF *A. flavus* were inoculated into 50 mL 100% seed liquid medium (sucrose 50 g/L, peptone 10 g/L, KH_2_PO_4_ 0.2 g/L, MgSO_4_·7H_2_O 0.2 g/L, Tween 60 15 g/L, pH 6). The seed fermentation liquid was obtained by shaking culture at 30 °C and 220 r/min for 24 h. Peanut meal is the waste material left over from harvesting peanut oil. For this formulation, the meal was crushed by a high-speed universal crusher until most of the sample passed through a sieve with a pore diameter of 1.0 mm and sterilized at 121 °C for 30 min. Sterile water was then added at a solid-liquid mass ratio of 7:3, which was a favorable condition for the growth of the biocontrol strain, and the moisture was mixed evenly. The seed fermentation liquid containing 18PAsp-zy1 was transferred to the mixed peanut meal medium at a mass ratio of 10%. The medium was cultured at 25 °C after being sealed with a piece of paper to prevent dirt from entering the box. The growth of the strain was observed every day until the spore concentration was 10^8^ colony-forming unit per gram (CFU/g). The peanut meal fertilizer was stored in the laboratory at room temperature and sealed for backup.

The spores used in the peanut coating agent were obtained after one week of incubation in soybean medium washed with sterile 0.2% Tween 20. The peanut coating agent was made by dissolving 1% *w*/*v* water-soluble starch, 0.5% *w*/*v* sodium alginate and 0.2% *w*/*v* glycerol in distilled water and then mixed with different concentrations of 18PAsp-zy1 spore suspension in a 2:1 volume ratio [48]. The concentrations of spores in the peanut coating agent were 10^8^ and 10^9^ CFU/mL for the low and high doses, respectively. The peanut coating agent was stored in sealed bottles at 4 °C in the laboratory and set aside.

In 2018, rice inoculum spores and peanut meal fertilizer were both spread on the soil by hand during the peanut flowering period. However, their dosages were different. The dosage of rice inoculum was 10 kg/ha with spore concentrations of 10^8^ and 10^9^ CFU/g for the respective low and high doses. The spore concentration in the peanut meal fertilizer was 10^8^ CFU/g in all cases, and the difference between the low and high doses was the amount of fertilizer, which was 10 and 20 kg/ha, respectively. The peanut coating agent was mixed well in the peanut seeds and then dried and sown.

In 2019, rice inoculated with non-AF *A. flavus* spores at rates of 7.5, 10 and 15 kg/ha were applied during the peanut flowering period.

### 5.3. Field Assays

The field experiments were conducted in 2018 from May to September in Zhengyang City in Henan Province, and June to October in Xiangyang City in Hubei Province. A piece of field (nearly 52 ha) was chosen in one city in each province. The management of the planting was under the unified responsibility of Shandong Luhua Group Corporation Limited (Yantai, China). Each field was divided into twenty-four equal-sized plots separated by 100 m fallow zones. Each individual plot measured 1250 m^2^ and was assigned a formulation treatment with a high dose or a low dose. The assignment of plots to treatments in each field was determined using a randomized complete block design (RCBD) [49]. The remaining plots were left untreated and served as controls. In each field, treatments were replicated three times.

A field (nearly 10 ha) was chosen to conduct the experiment in 2019 from May to September in Zhengyang City in Henan Province. The field was divided into twelve equal-sized plots separated by 100 m from each other. Each individual plot was measured 666 m^2^. The assignment of plots to treatments in each field was determined using a randomized complete block design (RCBD) [49]. The remaining plots were left untreated and served as controls. In the field, treatments were replicated three times.

Soil samples from each plot were collected before planting and at harvest using a five-point sampling method. A total of 100 g of soil samples were collected at each point at a depth of 2 cm. The obtained soil samples were mixed evenly and stored in a bag at 4 °C. At harvest, peanuts (300 g) were collected from each point; therefore, 1.5 kg was collected from each plot. The peanuts were evenly mixed and baked at 65 °C for 24 h. The moisture content was determined before the analysis of aflatoxins.

### 5.4. Distribution of Aspergillus flavus in Soil Samples

To detect the colonization of non-AF strains in the soil, the density of *A. flavus* and the proportion of non-AF strains in each plot before planting and at harvest were examined in this study. Ten grams of each soil sample was added to 90 mL of 0.1% sterile peptone solution [46]. The mixture was shaken at 30 °C and 220 r/min for 30 min. Then, 1 mL of the mixture was placed in a centrifuge tube with 9 mL of 0.1% peptone sterile water to prepare a 1:100 diluted sample. Finally, 10^−1^, 10^−2^, 10^−3^ soil dilutions were chosen for plating. One-hundred microliters of each dilution was evenly smeared on PDA plates, which was repeated three times for each dilution, and incubated at 30 °C. The growth of colonies was observed closely. A single colony suspected to be *A. flavus* was isolated and cultured on dichloran 18% glycerol agar (DG18: 31.6 g/L, Beijing Aoboxing Biotechnology Co. Ltd., Beijing, China) and transferred to *Aspergillus flavus* and *parasiticus* agar (AFPA: 45.6 g/L, Qingdao Hope Biotechnology Co., Ltd, Qingdao, China) to verify whether the colony was *A. flavus* or *A. parasiticus*. The selection of strains was random, and the inspection of each strain was subjected to the Markov process [50]. Ten strains of *A. flavus* on a 10^−2^ plate were randomly selected to identify their toxin-producing abilities using a PCR-RFLP method based on the *aflR* gene combined with a toxin production test [23]. Therefore, ninety strains of *A. flavus* were selected to identify their ability to produce aflatoxins in each treatment. A total of 1260 strains were identified in this study.

### 5.5. Aflatoxin Assessments

Aflatoxin B_1_ (AFB_1_) content was measured in peanuts collected at harvest from treated and untreated field plots. Additionally, AFB_1_ assessments were conducted on peanut samples collected after three months of normal storage in the warehouse of Luhua Group Corporation Limited (Yantai, China), which included a pre-storage drying period of three to five days. Another assay was conducted on peanut samples that underwent three months of storage at 30 °C under constant humidity (>90%). An immunoaffinity column extraction method and high-performance liquid chromatography (HPLC) analysis were performed according to Shotwell et al. [51] with slight modification.

Peanuts (1.5 kg) were dried and crushed, and then 20 g of each sample was transferred to an Erlenmeyer flask with 4 g of sodium chloride and 100 mL of extraction solution (70% methanol-water solution). The mixture was homogenized for three minutes followed by filtration with fast qualitative filter paper. Ten milliliters of filtrate was mixed with 20 mL of water and then filtered through microfiber filter paper. The filtrate was collected as a sample solution, of which 15 mL was purified by an AFB_1_ immunoaffinity column (Beijing Hua’an Maike Biotechnology Co., Ltd., Beijing, China), collected into a labelled liquid phase vial and stored at −20 °C.

Subsequently, the AFB_1_ content in each vial was analyzed by an Alliance e2695 HPLC system (Waters, Milford, DE, USA) equipped with a 2475 fluorescence detector (excitation 365 nm, emission 450 nm), an autosampler system and an improved photochemical reactor (AURA, New York, NY, USA, 230 Volt, 50 Hz, 8 Watt). Methanol and water were used as the mobile phase with an equal volumetric ratio at a speed of 0.5 mL/min. The limits of detection and quantification for AFB_1_ were 0.5 and 0.75 ng/mL, respectively.

### 5.6. Statistical Analysis

The total number of fungal colonies and *A. flavus* were calculated according to the Chinese National Standards GB4789.15-2016 [52]. The density of colonies in the soil was expressed as CFU/g. The incidence (%) of non-AF strains in each province was the ratio of the number of non-AF *A. flavus* in each treatment to the total number of *A. flavus* picked for each treatment in each province (90) according to Formula (1).

(1) $$\mathrm{Incidence}\;\mathrm{of}\;\mathrm{atoxigenic}\;\mathrm{strains}\left(\%\right)=\frac{\begin{array}{l} \mathrm{number}\;\mathrm{of}\;\mathrm{atoxigenic}\;A.\;flavus\;\\ \mathrm{in}\;\mathrm{each}\;\mathrm{treatment}\;\mathrm{in}\;\mathrm{each}\;\mathrm{province} \end{array}}{\begin{array}{l} \mathrm{total}\;\mathrm{number}\;\mathrm{of}\;A.\;flavus\;\mathrm{picked}\;\\ \mathrm{for}\;\mathrm{each}\;\mathrm{treatment}\;\mathrm{in}\;\mathrm{each}\;\mathrm{province}\; \end{array}}\times 100$$

The percentage reduction of aflatoxin B_1_ was obtained by comparing aflatoxins in treated fields with the value in the control field in the corresponding province according to Formula (2).

(2) $${\mathrm{Reduction}\;\mathrm{in}\;\mathrm{aflatoxin}\;B}_{1}\left(\%\right)=\frac{\begin{array}{l} {\mathrm{mean}\;\mathrm{aflatoxin}\;B}_{1}\;\mathrm{content}\;\mathrm{in}\;\mathrm{peanuts}\;\mathrm{in}\;\mathrm{untreated}\;\mathrm{field}\;-\\ {\mathrm{mean}\;\mathrm{aflatoxin}\;B}_{1}\;\mathrm{content}\;\mathrm{in}\;\mathrm{peanuts}\;\mathrm{in}\;\mathrm{treated}\;\mathrm{field} \end{array}}{{\mathrm{mean}\;\mathrm{aflatoxin}\;B}_{1}\;\mathrm{content}\;\mathrm{in}\;\mathrm{peanuts}\;\mathrm{in}\;\mathrm{untreated}\;\mathrm{field}}\times 100$$

Fungal density data and changes in AFB_1_ concentrations in peanuts were log-transformed before analysis. Statistical analysis was performed by one-way analysis of variance (ANOVA) followed by Fisher’s least significant differences (LSD) tests using SPSS Statistics 26.0 software (IBM Corporation, Armonk, NY, USA). A value of *p* < 0.05 was taken as the degree of significance.

## Figures and Tables

**Table 1:** Distribution of *A. flavus* (logCFU/g) and incidence (%) of non-AF strains from biocontrol-treated fields in 2018.

Treatment ^a^	Henan Province				Hubei Province			
	Soil before Planting		Soil at Harvest		Soil before Planting		Soil at Harvest	
	*A. flavus* (logCFU/g)	Non-AF (%)	*A. flavus* (logCFU/g)	Non-AF (%)	*A. flavus* (logCFU/g)	Non-AF (%)	*A. flavus* (logCFU/g)	Non-AF (%)
Untreated/Control	3.44	25.56 ± 10.18	3.88	23.33 ± 0	3.43	22.22 ± 1.92	4.06	26.67 ± 6.67
Rice: low dose	3.48	23.33 ± 3.33	4.64	96.67 ± 5.77	3.54	21.11 ± 5.09	4.62	94.44 ± 3.85
Rice: high dose	3.26	28.89 ± 1.92	4.15	94.44 ± 5.09	3.44	21.11 ± 5.09	4.87	94.44 ± 9.62
Meal: low dose	3.49	31.11 ± 1.92	3.82	75.56 ± 5.09	3.36	23.33 ± 6.67	3.00	58.89 ± 6.94
Meal: high dose	3.44	24.44 ± 1.92	3.85	73.33 ± 6.67	3.50	31.11 ± 5.09	3.88	71.11 ± 3.85
Coat: low dose	3.05	30.00 ± 3.33	3.87	41.11 ± 3.85	3.62	25.56 ± 8.39	2.81	41.11 ± 1.92
Coat: high dose	3.21	22.22 ± 3.85	4.31	43.33 ± 3.33	3.29	26.67 ± 5.77	4.35	42.22 ± 1.92

^a^ Rice grain colonized with biocontrol at low (10 kg/ha, with 10^8^ CFU/g of spores) or high (10 kg/ha, with 10^9^ CFU/g of spores) doses; peanut meal colonized with biocontrol at low (10 kg/ha, with 10^8^ CFU/g of spores) or high (20 kg/ha, with 10^8^ CFU/g of spores) doses; peanut coating agent colonized with biocontrol at low (10^8^ CFU/mL of spores) or high (10^9^ CFU/mL of spores) doses.

**Table 2:** Distribution of *A. flavus* (logCFU/g) and incidence (%) of non-AF strains from biocontrol-treated fields in 2019.

Treatment ^a^	Soil before Planting		Soil at Harvest	
	*A. flavus* (logCFU/g)	Non-AF (%)	*A. flavus* (logCFU/g)	Non-AF (%)
Untreated/Control	3.46	26.67 ± 6.67	3.86	24.44 ± 3.85
Rice: low dose	3.49	28.89 ± 5.09	4.07	87.78 ± 1.92
Rice: medium dose	3.48	26.67 ± 8.82	4.38	94.44 ± 6.94
Rice: high dose	3.44	27.78 ± 6.94	4.35	92.22 ± 6.94

^a^ Rice grain colonized with biocontrol at low (7.5 kg/ha, with 10^8^ CFU/g of spores), medium (10.0 kg/ha, with 10^8^ CFU/g of spores) or high (15.0 kg/ha, with 10^8^ CFU/g of spores) doses.

**Table 3:** Reductions in AFB_1_ content based on biocontrol-treated peanut fields in 2018.

Treatment ^a^	Henan Province		Hubei Province	
	Aflatoxin B_1_ Content (μg/kg)	Reduction (%)	Aflatoxin B_1_ Content (μg/kg)	Reduction (%)
Untreated/Control	41.35 ± 3.80	-	62.29 ± 10.07	-
Rice: low dose	10.61 ± 3.77	74.34	14.57 ± 9.36	76.61
Rice: high dose	7.48 ± 1.18	81.90	12.41 ± 4.59	80.07
Meal: low dose	12.85 ± 1.29	68.92	23.54 ± 2.29	62.21
Meal: high dose	10.97 ± 0.97	73.48	21.47 ± 1.47	65.54
Coat: low dose	23.02 ± 2.74	44.33	34.61 ± 0.91	44.44
Coat: high dose	19.85 ± 0.94	52.00	54.10 ± 4.29	13.15

^a^ Rice grain colonized with biocontrol at low (10 kg/ha, with 10^8^ CFU/g of spores) or high (10 kg/ha, with 10^9^ CFU/g of spores) doses; peanut meal colonized with biocontrol at low (10 kg/ha, with 10^8^ CFU/g of spores) or high (20 kg/ha, with 10^8^ CFU/g of spores) doses; peanut coating agent colonized with biocontrol at low (10^8^ CFU/mL of spores) or high (10^9^ CFU/mL of spores) doses.

## Data Availability

Not applicable.
